# Metronomic 5-Fluorouracil Delivery Primes Skeletal Muscle for Myopathy but Does Not Cause Cachexia

**DOI:** 10.3390/ph14050478

**Published:** 2021-05-17

**Authors:** Dean G. Campelj, Cara A. Timpani, Tabitha Cree, Aaron C. Petersen, Alan Hayes, Craig A. Goodman, Emma Rybalka

**Affiliations:** 1 Institute for Health and Sport, Victoria University, Melbourne, VIC 8001, Australia; dean.campelj@live.vu.edu.au (D.G.C.); cara.timpani@vu.edu.au (C.A.T.); tabitha.cree@live.vu.edu.au (T.C.); aaron.petersen@vu.edu.au (A.C.P.); alan.hayes@vu.edu.au (A.H.); 2 Australian Institute for Musculoskeletal Science (AIMSS), Inherited and Acquired Myopathy Program, Victoria University, St Albans, VIC 3021, Australia; 3 Department of Medicine-Western Health, Melbourne Medical School, The University of Melbourne, Melbourne, VIC 3021, Australia; 4 Centre for Muscle Research (CMR), Department of Physiology, The University of Melbourne, Parkville, VIC 3010, Australia

**Keywords:** chemotherapy, cachexia, 5-fluorouracil, skeletal muscle, p38, NF-B, dystrophin, desmin

## Abstract

Skeletal myopathy encompasses both atrophy and dysfunction and is a prominent event in cancer and chemotherapy-induced cachexia. Here, we investigate the effects of a chemotherapeutic agent, 5-fluorouracil (5FU), on skeletal muscle mass and function, and whether small-molecule therapeutic candidate, BGP-15, could be protective against the chemotoxic challenge exerted by 5FU. Additionally, we explore the molecular signature of 5FU treatment. Male Balb/c mice received metronomic tri-weekly intraperitoneal delivery of 5FU (23 mg/kg), with and without BGP-15 (15 mg/kg), 6 times in total over a 15 day treatment period. We demonstrated that neither 5FU, nor 5FU combined with BGP-15, affected body composition indices, skeletal muscle mass or function. Adjuvant BGP-15 treatment did, however, prevent the 5FU-induced phosphorylation of p38 MAPK and p65 NF-B subunit, signalling pathways involved in cell stress and inflammatory signalling, respectively. This as associated with mitoprotection. 5FU reduced the expression of the key cytoskeletal proteins, desmin and dystrophin, which was not prevented by BGP-15. Combined, these data show that metronomic delivery of 5FU does not elicit physiological consequences to skeletal muscle mass and function but is implicit in priming skeletal muscle with a molecular signature for myopathy. BGP-15 has modest protective efficacy against the molecular changes induced by 5FU.

## 1. Introduction

Colorectal cancer (CRC) is a significant contributor to worldwide morbidity and mortality, with approximately 1.9 million new CRC cases and 900,000 CRC-related deaths reported in 2020 (GLOBOCAN 2020) [1]. The underlying causes of CRC progression and mortality are multi-faceted and still being discovered. A key risk factor that complicates responsivity to anti-cancer treatment, and, therefore survivability, is the metabolic wasting syndrome, cachexia, which is prevalent in ~45% of CRC patients [2]. Cachexia is characterised by the ongoing loss of skeletal muscle mass, with or without loss of fat mass, where patients are non-responsive to nutritional intervention and manifest progressive functional impairment [3]. Clinically, skeletal muscle atrophy and poor recovery from the loss of skeletal muscle mass have been established as two prominent prognostic factors of mortality in cachectic cancer patients [4]. Recently, anti-cancer treatment, i.e., chemotherapy, has surfaced as a key contributor to the progression of cachexia, with emerging literature suggesting that chemotherapy can induce skeletal muscle mass loss and dysfunction (skeletal myopathy) [5,6] with lifelong impact [7].

Chemotherapy is a mainstay of advanced CRC treatment strategies and is used complementarily to surgical tumour resection or in advanced staging due to metastasis or resistance to radiotherapy. The anti-metabolite, 5-fluorouracil (5FU), is primarily utilized in the treatment of advanced CRC as a backbone constituent of multi-agent regimens, such as FOLFIRI [5FU, leucovorin (LV) and irinotecan (IRI)] and FOLFOX [5FU, LV and oxaliplatin (OXA)] (for extensive review, see [8]). 5FU administration can elicit significant side-effects including, but not limited to, cardiotoxicity, gastrointestinal toxicity, neurotoxicity and pan-cytopenia [9], with lean mass suggested to be an independent determinant of dose-limiting toxicity from 5FU-based regimens [10]. Barreto et al. have demonstrated that the 5FU-based FOLFIRI regimen induces a cachectic phenotype in cancer-free mice, underscored by the loss of body and lean mass, and skeletal myopathy [11]. This was in contrast with the FOLFOX regimen, which did not induce any of these effects [11]. These data suggest that IRI is more impactful on the musculoskeletal system than OXA, and to this effect, we have recently demonstrated that IRI causes acute cachexia, lean tissue wasting and skeletal muscle dysfunction [12]. However, whether 5FU also induces cachectic wasting and skeletal myopathy, independent from the other constituents in the FOLFIRI regimen, is unclear [13,14]. Such information is of clinical importance so that the risk of cachexia during administration of FOLFIRI and other 5FU-based regimens can be predicted and clinically managed.

Chemotherapy-induced skeletal muscle wasting is underlined by compromised proteostasis in favour of protein degradation [6]. It is characterised by a molecular signature of myopathy involving the activation of the stress-inducible mitogen-activated protein kinases (MAPK), p38 and ERK1/2 [11], and key transcription factor for inflammatory genes, nuclear factor-B (NF-B) [15]. Since MAPK phosphorylation can interact with NF-B phosphorylation during oxidative stress [16], it is suggested that there is a link between these two target pathways, leading to the induction of a pro-catabolic environment [17,18]. Specifically, 5FU is a potent activator of p38 MAPK signalling in vitro [19], and in vivo evidence from the 5FU-based FOLFIRI regimen supports the same—increased phosphorylation of p38 MAPK associated with enhanced oxidative stress and reduced mitochondrial quality control signalling [11]. These data are consistent with findings from our laboratory, where 5FU potentiated mitochondrial superoxide production and reduced mitochondrial viability in C2C12 skeletal muscle cells [20]. The FOLFIRI regimen also reduces mitochondrial content, which is associated with abnormalities in muscle morphology at the level of the sarcomere [11,21]. This was of interest to our group since we have recently shown that the chemotherapeutic agent, IRI, compromised cytoskeletal stability through reducing dystrophin protein expression [12]. While the mechanism underlying the effect of chemotherapy on skeletal muscle cytoskeletal composition is still undefined, it may be an event related to chemotherapy-induced NF-B activation [15], suggesting that NF-B activity plays a role in the decay of structural integrity of muscles [22]. Therefore, in this study, our first aim was to characterise the effect of 5FU treatment on skeletal muscle mass and function, and to investigate the underlying molecular mechanisms with emphasis on the potential connection between p38 MAPK and NF-B signalling, mitochondrial dynamics and structural cytoskeletal proteins.

There is estimated to be 3.15 million new CRC cases worldwide in 2040 [1], highlighting the growing burden of cancer and the current need for novel therapeutic strategies to support current anti-cancer treatments and promote survivability. BGP-15 is a nicotinic amidoxime derivate and small-molecule therapeutic candidate which acts as a cytoprotectant through the inhibition of poly (ADP) ribose polymerase -1 (PARP-1) and co-induction of heat shock protein-70 (HSP-70) [23,24]. BGP-15-mediated PARP-1 inhibition and HSP-70 co-induction are associated with improved mitochondrial content, function and oxidative capacity [25,26]. However, BGP-15 has also been shown to elicit mitoprotection independent of these targets, highlighting its pleiotropic efficacy [27]. Consequently, BGP-15 has promise in the treatment of a range of skeletal myopathies, including diabetes, Duchenne Muscular Dystrophy and ventilation-induced diaphragm dysfunction (for extensive review see [28]). We have evaluated the adjuvant therapeutic potential of BGP-15 to protect against the induction of skeletal myopathy from chemotherapeutic agents IRI and OXA [12,29], with mixed efficacy. However, since 5FU is a potent activator of p38 MAPK, and BGP-15 has been shown to inhibit pan-MAPK activity [30], the potential for BGP-15 to be more efficacious when administered with 5FU is substantial. Thus, our secondary aim was to evaluate the protective efficacy of BGP-15 adjuvant therapy against 5FU-induced skeletal myopathy and investigate the underlying mechanisms through which BGP-15 functions as a p38 MAPK inhibitor in a chemotoxic environment.

## 2. Results

### 2.1. Assessment of Body Composition Indices, Skeletal Muscle Mass and Function

**Figure 1:**
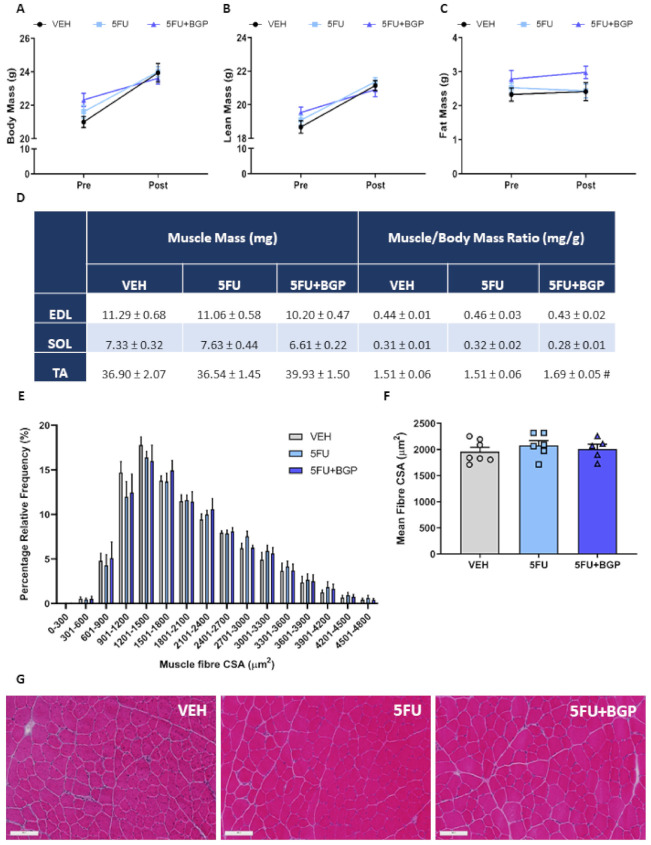
The effect of 5-fluorouracil (5FU) and 5FU with BGP-15 (5FU+BGP) treatment on body composition and muscle size indices. Body composition parameters were measured and presented as pre- and post-treatment data points for (**A**) body, (**B**) lean and (**C**) fat mass. (**D**) Hindlimb skeletal muscles extensor digitorum longus (EDL), soleus (SOL), and tibialis anterior (TA) were weighed post-treatment and data presented as raw mass and muscle to body mass ratios (# *p* = 0.07; compared to 5FU). TA cross-sections were H&E-stained and underwent histological fibre size analysis with data presented as (**E**) percentage relative frequency distribution of the muscle fibre cross-sectional area (CSA) and (**F**) mean muscle fibre CSA. (**G**) Representative images of H&E-stained TA cross-sections are displayed. Scale bar = 100 m. *n* = 7–8 for body composition indices; *n* = 4–8 for muscle weights; *n* = 5–7 for histology. Data are mean ± SEM.

**Figure 2:**
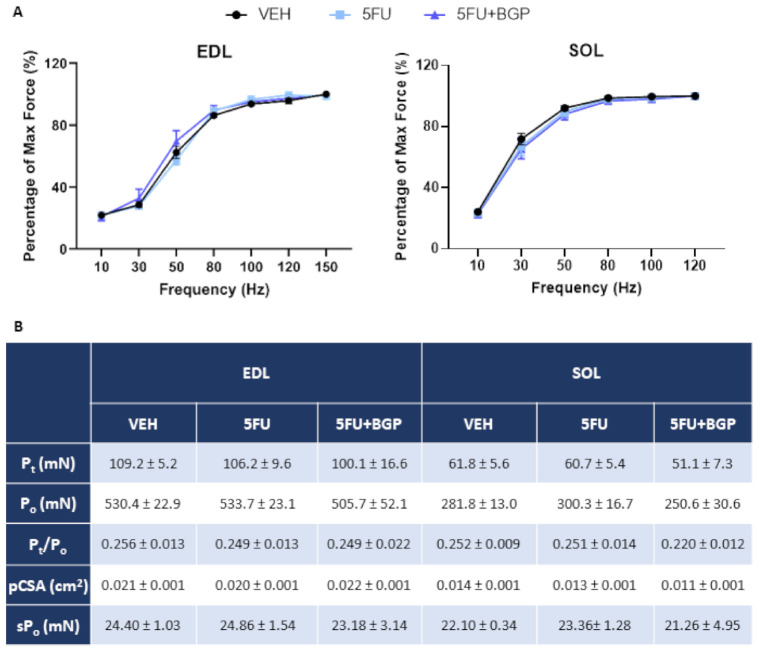
The effect of 5-fluorouracil (5FU) and 5FU with BGP-15 (5FU+BGP) treatment on skeletal muscle contractile function. Extensor digitorum longus (EDL) and soleus (SOL) muscles underwent ex vivo assessment of contractile functional properties, with (**A**) force–frequency relationships and (**B**) force production characteristics analyzed, including; Peak twitch force (P_t_), Absolute tetanic force production (P_o_), twitch to tetanus ratio (P_t_/P_o_), physiological cross-sectional area (pCSA) and Specific force production (sP_o_). *n* = 4–8 for ex vivo contractile function. Data are mean ± SEM.

To explore the potential contribution of 5FU treatment on the induction of cachexia and efficacy of 5FU+BGP treatment, we examined a suite of body composition and skeletal muscle size indices. Interestingly, 5FU did not inhibit growth nor reduce body, lean or fat mass, and subsequently, 5FU+BGP also did not affect these parameters (Figure 1A–C; Table S1). Consistent with body composition data, raw skeletal muscle mass and skeletal muscle mass to body mass ratios for EDL, SOL and TA muscles were not significantly different between treatment groups (Figure 1D), although, 5FU+BGP displayed a trend to increase the TA to body mass ratio compared to 5FU treatment (*p* = 0.07, Figure 1D). Organ mass also remained unchanged between treatment groups (Table S1). We then undertook muscle fibre size analysis in TA cross-sections, with no significant differences found between treatment groups for the relative frequency percentage of muscle fibre cross-sectional areas (CSA) (Figure 1E,G)) nor the mean fibre CSA (Figure 1F,G). Next, we assessed the ex vivo contractile function of EDL and SOL muscles. Neither 5FU, nor 5FU+BGP treatment affected skeletal muscle functional parameters of EDL or SOL, with no change observed in force–frequency relationships (Figure 2A), force production characteristics, i.e., P_t_, P_o_, P_t_/P_o_ and sP_o_ (Figure 2B).

### 2.2. Assessment of Cytoskeletal Structural Protein Expression

**Figure 3:**
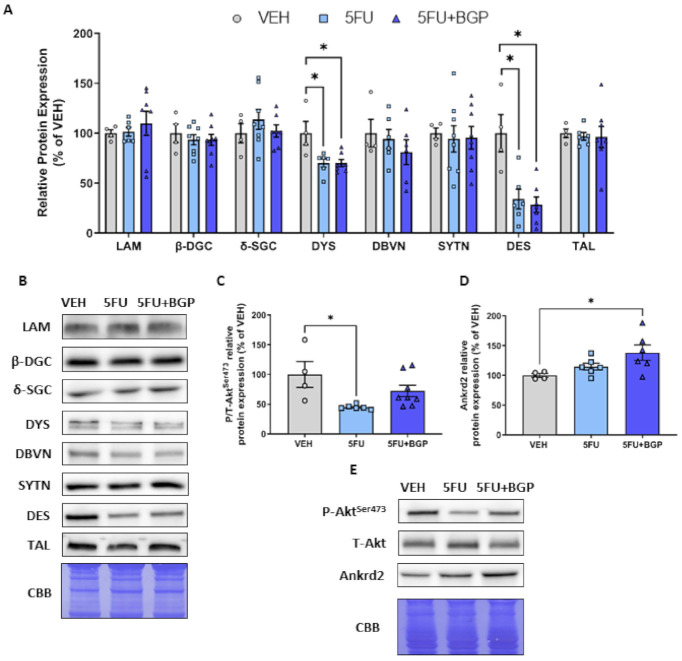
The effect of 5-fluorouracil (5FU) and 5FU with BGP-15 (5FU+BGP) treatment on expression of cytoskeletal structural proteins. Western blotting experiments were undertaken in tibialis anterior (TA) muscle homogenate, with samples probed for (**A**,**B**) cytoskeletal structural proteins including; laminin (LAM), -dystroglycan (-DGC), -sarcoglycan (-SGC), dystrophin (DYS), dystrobrevin (DBVN), syntrophin (SYTN), desmin (DES) and talin (TAL). (**C**) Phosphorylated (Ser473) and total Akt were probed for as an indicator of mammalian target of rapamycin Complex 2 (mTORC2) activity. (**D**) Ankrd2 was probed for as a marker of mechano-sensitvit. (**E**) Representative images for phosphorylated Akt^Ser473^, total Akt and Ankrd2 displayed. Protein expression was normalised to total protein derived from Coomassie Brilliant Blue (CBB) staining and presented relative to vehicle (VEH) control group. * = *p* < 0.05; *n* = 4 for VEH and *n* = 6–8 for 5FU and 5FU+BGP groups for Western blotting. Data are mean ± SEM.

Previously, we have shown an association between skeletal muscle dysfunction and reduced expression of proteins associated with the dystrophin-associated protein complex (DAPC) induced by IRI treatment [12]. Thus, we investigated these parameters in the context of this study. 5FU treatment induced a reduction in the abundance of the cytoskeleton proteins, desmin and dystrophin (*p* < 0.05, Figure 3A,B), and 5FU+BGP treatment did not protect against these changes. There was no impact of 5FU, nor 5FU+BGP, treatment on the protein expression of additional cytoskeletal structural proteins laminin, -dystroglycan, -sarcoglycan, dystrobrevin, syntrophin and talin (Figure 3A,B). Next, we explored the effect of 5FU and 5FU+BGP treatment on Akt^Ser473^ phosphorylation, an established target of Mechanistic Target of Rapamycin Complex 2 (mTORC2) [31]. mTORC2 activity has been shown to be involved in the regulation of plasma membrane homeostasis and cytoskeletal organization [32]. Thus, we were interested in mTORC2 activity in the context of the reduced expression of desmin and dystrophin—the phosphorylation of Akt ^Ser473^ is indicative of mTORC2 activation. We showed that 5FU treatment reduced the phosphorylation of Akt^Ser473^ compared to VEH (*p* < 0.05, Figure 3C,E), while 5FU+BGP-15 treatment did not alter the 5FU-induced changes to Akt^Ser473^ signalling (Figure 3C).

Given our evidence that 5FU reduced desmin and dystrophin expression and Akt^Ser473^ phosphorylation, we investigated potential changes to the mechano-sensitive protein, ankyrin repeat domain protein 2 (Ankrd2), a member of Muscle Ankyrin Repeat Domain (MARP) family of titin filament-based stress response proteins [33]. Ankrd2 could be a target of chemotherapy within skeletal muscle since it is responsive to oxidative stress, a key mechanism of chemotherapy-induced muscle wasting. We demonstrated that 5FU treatment did not affect Ankrd2 protein expression (Figure 3D,E). However, 5FU+BGP treatment increased Ankrd2 expression relative to VEH (*p* < 0.05, Figure 3D,E). These data suggest that BGP-15 may be promoting a futile adaptive stress response that enhances mechano-sensitivity, despite the perturbed cytoskeletal environment. Full-length blots are provided in Figure S1 and densitometry summary data are provided in Table S2.

### 2.3. Assessment of Skeletal Muscle Stress Signalling

**Figure 4:**
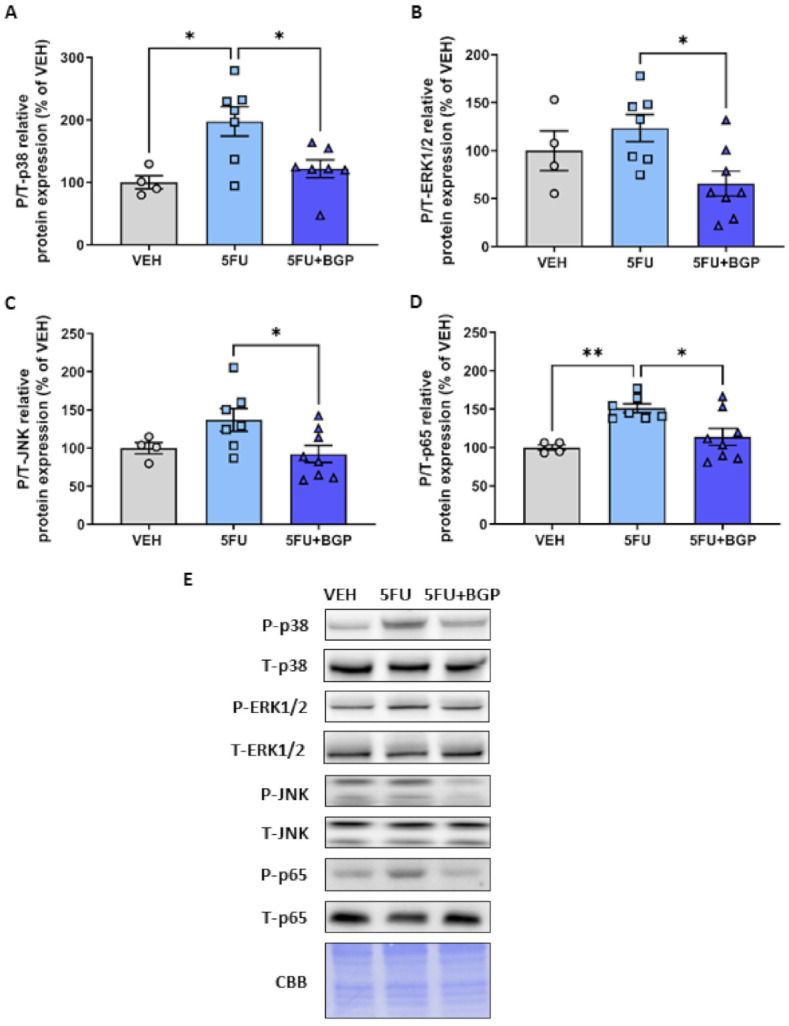
The effect of 5-fluorouracil (5FU) and 5FU with BGP-15 (5FU+BGP) treatment on molecular markers of cellular stress. Western blotting experiments were undertaken on tibialis anterior muscle homogenate. Samples were probed for (**A**) phosphorylated (Thr180/Tyr182) and total p38, (**B**) phosphorylated (Thr202/Tyr204) and total ERK1/2, (**C**) phosphorylated (Thr183/Tyr185) and total JNK, (**D**) phosphorylated (Ser536) and total NF-B subunit protein p65. Data are presented as phosphorylated to total protein ratios and normalised to total protein derived from Coomassie Brilliant Blue (CBB) staining and expressed as a relative percentage of the vehicle (VEH) control grou (**E**) Western blotting and CBB representative images are displayed. * = *p* < 0.05; *n* = 4 for VEH and *n* = 6–8 for 5FU and 5FU+BGP groups. Data are mean ± SEM. ** = *p* < 0.01.

Since 5FU has been shown to potently activate p38-MAPK signalling [19], we wanted to assess the effect of 5FU and 5FU+BGP treatment on p38-MAPK and other molecular markers of skeletal muscle stress signalling. Consistent with the literature, 5FU treatment increased the phosphorylation of p38; (*p* < 0.05, Figure 4A,E). However, the phosphorylation of other MAPK family members, ERK1/2 or JNK, were not affected (Figure 4B,C,E). Importantly, 5FU+BGP-15 treatment completely mitigated the 5FU-induced increase in p38 phosphorylation (*p* < 0.05, Figure 4A,E). Furthermore, and consistent with BGP-15${}^{\prime }$s reported role as a suppressor of MAPK activity [28], 5FU+BGP-15 reduced basal levels of ERK1/2 and JNK phosphorylation relative to 5FU treatment (*p* < 0.05, Figure 4B,C,E). 5FU also increased the phosphorylation of the p65 subunit of NF-B, a transcriptional mediator of pro-inflammatory genes [34], and this was inhibited by co-treatment with BGP+15 (*p* < 0.05, Figure 4D,E). Full-length blots are provided in Figure S2 and densitometry summary data are provided in Table S2.

### 2.4. Assessment of Skeletal Muscle Oxidative Capacity and Mitochondrial Dynamics

**Figure 5:**
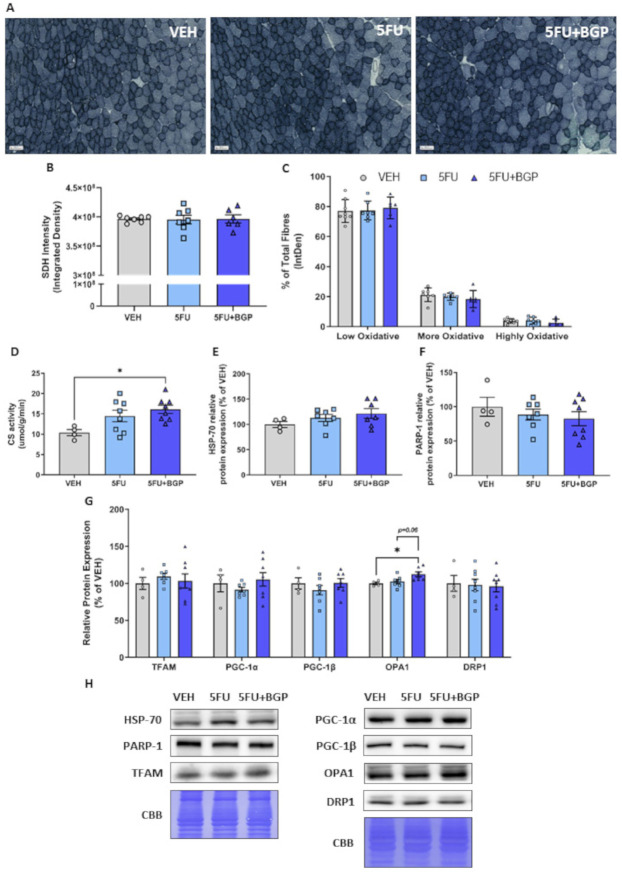
The effect of 5-fluorouracil (5FU) and 5FU with BGP-15 (5FU+BGP) treatment on skeletal muscle oxidative capacity and mitochondrial dynamics signalling. Succinate dehydrogenase (SDH) staining was performed on tibialis anterior (TA) cross-sections. (**A**) SDH representative images displayed, with data presented as (**B**) overall SDH intensity and (**C**) SDH intensity separated based on oxidative fibre phenotype, i.e., low oxidative, more oxidative and highly oxidative. TA muscle homogenate was analyzed for (**D**) citrate synthase (CS) activity, as a marker of mitochondrial density. Further, TA homogenate was utilized in Western blotting experiments with samples probed for (**E**) HSP-70, (**F**) PARP-1 and (**G**) a suite of proteins related to mitochondrial dynamics including; TFAM, PGC-1, PGC-1, OPA1 and DRP1. Protein expression was normalised to total protein derived from Coomassie Brilliant Blue (CBB) staining and presented relative to vehicle (VEH) control group. (**H**) Western blotting and CBB representative images are displayed. * = *p* < 0.05; *n* = 6–8 for SDH histology; *n* = 4 for VEH and *n* = 6–8 for 5FU and 5FU+BGP groups for CS activity and Western blotting. Data are mean ± SEM.

Next, we examined skeletal muscle oxidative capacity and markers of mitochondrial dynamics, as previously we have shown in vitro that 5FU reduces mitochondrial viability in C2C12 myoblast and myotubes [20]. Further, BGP-15 adjunct therapy alongside the anti-cancer agent, IRI, increased mitochondrial density and oxidative metabolism [12]. Interestingly, skeletal muscle oxidative capacity, as measured histologically via SDH staining, was not affected by 5FU and 5FU+BGP treatment (Figure 5A–C). 5FU treatment did not affect mitochondrial content as measured by CS activity (Figure 5D), although 5FU+BGP-15 treatment enhanced CS activity compared to VEH (*p* < 0.05, Figure 5D). We thought this may occur through the proposed mechanistic targets of BGP-15, i.e., HSP-70 co-induction or PARP-1 inhibition [23,24], which augment mitoprotection [25,26]. However, we demonstrated that there was no change to the protein expression of HSP-70 or PARP-1 from 5FU or 5FU+BGP treatment (Figure 5E,F,H). Next, we evaluated molecular markers of mitochondrial dynamics, including mitochondrial biogenesis, i.e., mitochondrial transcription factor A (TFAM) and peroxisome proliferator-activated receptor-gamma coactivator-1 (PGC-1) isoforms and , and mitochondrial fusion, i.e., optic atrophy 1 (OPA1), and fission, i.e., dynamin-related protein 1 (DRP1). There were no changes to TFAM, PGC-1, PGC-1 and DRP-1 from 5FU or 5FU+BGP treatment (Figure 5G,H), but there was a significant increase in OPA-1 protein expression from 5FU+BGP treatment compared to VEH, with no change pertaining to 5FU treatment (Figure 5G,H). This suggests that the increase in mitochondrial content with 5FU+BGP may, in part, be a result of enhanced mitochondrial fusion, a reported pleiotropic effect of BGP-15 [35]. Full-length blots are provided in Figure S2.

### 2.5. Assessment of HSP-70 Expression and Cell Viability in C2C12 Myotubes

**Figure 6:**
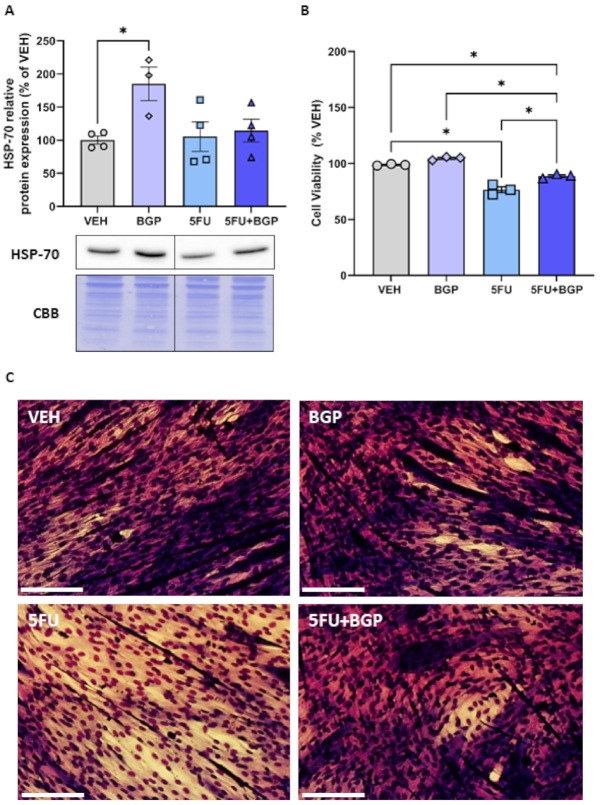
The effect of BGP-15 (BGP), 5-fluorouracil (5FU) and 5FU with BGP (5FU+BGP) treatment on HSP-70 expression and cell viabilityC2C12 myotubes were treated with BGP, 5FU and 5FU with BGP before being prepared as lysates for Western blotting experiments. (**A**) C2C12 lysates were then probed for HSP-70, with protein expression normalised to total protein derived from Coomassie Brilliant Blue (CBB) staining data and data presented as a percentage of the vehicle control group (VEH). (**B**) Cell viability was analyzed via the resazurin cell viability assay, with data presented as a percentage of VEH. (**C**) Representative images for the cell viability assay are displayed. Scale bar = 100 m. * = *p* < 0.05; *n* = 3–4 for all C2C12 experiments. Data are mean ± SEM.

Given that we saw no change in HSP-70 protein expression in vivo, we wanted to understand some of the potential reasons for these data. Thus, we pursued an in vitro investigation utilising differentiated C2C12 myotubes to delineate the effect of BGP-15 treatment on HSP-70 expression in both a standard and 5FU-induced chemotoxic medium. Importantly, we demonstrated that BGP-15 increased HSP-70 protein expression compared to VEH (*p* < 0.05, Figure 6A). However, in a 5FU-induced chemotoxic medium, the induction of HSP-70 by BGP-15 was blunted, thus remaining unchanged from VEH (Figure 6A). Next, we assessed cell viability, through resazurin staining, confirming that 5FU treatment induced a chemotoxic environment, compared to VEH (*p* < 0.05, Figure 6B,C). BGP-15 co-treatment displayed a modest improvement in cell viability, compared to 5FU (*p* < 0.05, Figure 6B,C). However, it was still significantly reduced compared to both VEH and BGP control groups (*p* < 0.05, Figure 6B,C). These data highlight that BGP-15 elicits pleiotropic cytoprotection, that is in part, independent of HSP-70 induction, which is likely inhibited when given alongside 5FU. These data support our in vivo data, where there was also no effect of BGP-15 on HSP-70 expression. Full-length blots are provided in Figure S3.

## 3. Discussion

The major finding of this study is that metronomic delivery of 5FU reduced the expression of key cytoskeletal proteins, desmin and dystrophin and increased the phosphorylation of p38 MAPK and NF-B, events that are evident in other models of muscle wasting. Despite this, 5FU did not impact body composition indices or alter whole muscle mass or muscle fibre size. While our data suggest that these 5FU-mediated events are not sufficient to elicit a myopathic phenotype, they may, prime the muscle to be more susceptible to adverse effects of other constituents of multi-agent regimens, such as LV and IRI for FOLFIRI or LV and OXA for FOLFOX. Furthermore, this study found that, while BGP-15 co-therapy did not alter the 5FU-induced decrease in cytoskeletal protein abundance, it mitigated the increase in p38 MAPK and NF-B phosphorylation, which was associated with improved mitochondrial content and fusion dynamics. Together these data suggest that while 5FU primes the muscle for myopathy, BGP-15 has pleiotropic cytoprotectant functions that protect against the activation of this molecular stress signature.

5FU treatment reduced the expression of key cytoskeletal proteins, desmin, an intermediate filament that provides stability to sarcomeres, and dystrophin, a large protein that connects the actin cytoskeleton to the sarcolemma, with the potential to compromise the structural organization of skeletal muscle and alter intracellular signalling. Previously, we have demonstrated that chemotherapeutic agent, IRI, also reduces dystrophin expression, which was associated with reduced Akt^Ser473^ phosphorylation, a direct substrate of kinase mTORC2 [12,31]. Phosphorylation of Akt^Ser473^ is a putative indicator of plasma membrane homeostasis and cytoskeletal organization [32]. As such, our finding of reduced Akt^Ser473^ phosphorylation concomitant with a reduced desmin and dystrophin protein expression, suggests that 5FU may have disrupted sarcolemmal homeostasis, cytoskeletal organization and intracellular signalling. Consistent with our previous findings, BGP-15 adjuvant therapy was not protective against the chemotherapy-induced reduction in the expression of cytoskeletal proteins [12]. Desmin and dystrophin-related myopathies are often associated with debilitating dysfunction [36,37] and myopathy is compounded in desmin and dystrophin double-knockout mice, which manifest a remarkable dystrophic phenotype with profound deterioration of sarcomere organisation and Z-line alignment [38]. Our data suggest that a critical level of desmin and dystrophin loss must occur before there is evidence of functional and structural alterations. Indeed, Baretto et al. showed that when administered over a longer duration (twice a week for 5 consecutive weeks), the 5FU-based FOLFIRI regimen reduced skeletal muscle function concurrent with aberrant skeletal muscle morphology [11], highlighting a temporal component to observing loss of function from metronomic delivery of chemotherapeutic agents. It would be of interest in future studies to evaluate the expression of key cytoskeletal proteins and sarcomere morphology in response to 5FU treatment longitudinally, to determine whether the muscles can recover or whether they retain a propensity for myopathy, which may be exacerbated, for example, by mechanical stressors such as exercise. Interestingly, we demonstrated novel evidence that BGP-15 adjuvant therapy promotes the expression of mechano-sensitive *MARP* family member, Ankrd2 [39]. We hypothesize that BGP-15 is escalating an adaptive stress response targeting tension dynamics by enhancing mechano-sensitivity within the reduced desmin and dystrophin environment. Since Ankrd2 is localized to titin filaments and Ankrd2 abundance is similarly increased in a conditional titin knockout mouse model [40], it is possible that increased mechano-sensitivity and remodelling of titin dynamics are an additional pleiotropic effect of BGP-15, which, although not warranted in this study because 5-FU did not impact contractile function, contributes to its protective effect against loss of muscle function with other chemotherapeutic agents [12].

Chemotherapeutic agents, irrespective of class, induce the activation of p38 MAPK signalling as a function of their cytotoxic nature [41]. We demonstrated that 5FU treatment increased the phosphorylation of p38 MAPK in skeletal muscle, which was consistent with findings from Barreto et al. where 5FU, administered as a constituent of the FOLFIRI chemotherapy regimen, led to enhanced p38 MAPK activity [11]. Contrary to the FOLFIRI regimen, neither ERK1/2 nor JNK phosphorylation were increased by 5FU treatment [11]. MAPKs have been shown to exhibit specific time- and dose-dependent profiles during oxidative stress in C2C12 myoblasts [16]. Thus, we cannot rule out that ERK1/2 and JNK could have been activated at an earlier timepoint. Additionally, it is well acknowledged that p38 MAPK is positively associated with the NF-B complex, with both targets activated in response to oxidative stress [16] and mediators of inflammatory cytokine production [19,42], although NF-B signalling is purported to have a greater role in skeletal muscle wasting conditions (for extensive review [34]). Indeed, we showed that 5FU-induced p38 MAPK activation was associated with an increase in the phosphorylation of NF-B subunit protein, p65, an event depicting transactivation of NF-B, which is essential for enhancing its transcriptional activity [43]. Damrauer et al. similarly demonstrated that anti-cancer agent, cisplatin, enhances NF-B transcriptional activity through stimulating the DNA-heterodimerization of subunits p50 and p65 in C2C12 myotubes [15]. However, chemotherapy-induced NF-B activity in vitro reduced myotube diameter [15], while in the current study, we saw no evidence of skeletal muscle atrophy. This suggests the magnitude and persistency of NF-B activity is important for atrophy induction, as the metronomic regimen used in our study was protective of skeletal muscle mass. Interestingly, BGP-15 mitigated the 5FU-induced increase in the phosphorylation of p38 MAPK and NF-B subunit protein p65 in our study. Further, BGP-15 reduced the phosphorylation of MAPKs ERK 1/2 and JNK relative to 5FU. This is consistent with findings from Sarszegi et al., where BGP-15 suppressed MAPK phosphorylation, which was enhanced during imatinib (tyrosine kinase inhibitor)-induced cardiotoxicity [30]. While it is unsurprising that BGP-15 acted as a repressor of MAPK activity, it is novel that BGP-15 also inhibited the 5FU-induced activation of NF-B. These data could suggest a negative feedback mechanism, since 5FU-induced p38 MAPK phosphorylation in vitro has previously been shown to enhance the production of inflammatory cytokines, IL-6, TNF- and IL-1 [19], which are known stimulants of NF-B activity [42]. Future studies are required to confirm that 5FU-induced phosphorylation of p38 MAPK and NF-B subunit protein, p65, enhances inflammatory cytokine production and that BGP-15 has an anti-inflammatory effect in this context.

In this study, we observed no evidence of 5FU-induced alterations to oxidative capacity, mitochondrial content or mitochondrial dynamics signalling, suggesting that 5FU may require other cytotoxic-agents (e.g., the FOLFIRI regimen [11]) to perturb mitochondrial activity in vivo. This is in contrast to in vitro models where we have shown that 5FU induces mitochondrial stress in cultured C2C12 myoblasts and myotubes [20], where direct and persistent interaction between 5FU and muscle cells appears more impactful. Consistent with previous findings, we demonstrated that BGP-15 increases mitochondrial density when administered alongside chemotherapy [12]. However, in contrast, we did not observe a BGP-15-induced inhibition of PARP-1 expression in this study. Further, we did not detect a change in HSP-70 expression in vivo at the time of tissue collection, although, we did find that the addition of BGP-15 to 5FU-treated C2C12 myotubes blunted the induction of HSP-70. We hypothesize that 5FU enhances the production of pro-inflammatory cytokines [19], which have been shown to act as repressors to heat shock factor-1 transcription, inhibiting the HSP-70 adaptive stress response [44,45]. This has led our laboratory, and others, to postulate that BGP-15 acts as a cytoprotectant in response to chemical or disease-induced stress, with the underlying protective mechanisms inconsistent and largely dependent on the challenge elicited by a given stressor [27,46]. It is considered that BGP-15 may elicit cytoprotection through pleiotropic mechanisms that regulate mitochondrial quality control [47]. Indeed, we showed that BGP-15 adjuvant therapy did not affect mitochondrial biogenesis or fission signalling but did consistently promote mitochondrial fusion signalling through increased OPA1 expression (compared to VEH), which was associated with enhanced mitochondrial density.

An important, yet paradoxical, finding of this study was that 5FU treatment did not elicit any consequences on body composition indices, skeletal muscle mass or function, despite the alterations to skeletal muscle stress signalling (i.e., p38 and NF-B phosphorylation) that are usually associated with the induction of chemotherapy-induced cachexia and skeletal myopathy [11,15]. The lack of change to these physiological parameters from 5FU treatment may be due to the metronomic delivery of chemotherapy, which typically elicits less systemic toxicity than administration of single maximum tolerable dose (MTD) bolus or sequential daily treatments [48,49]. Metronomic delivery could activate an adaptive hormetic response which prevents the induction of skeletal myopathy in the face of low-grade stress signalling. Interestingly, when comparing to models that deliver 5FU sequentially in a shorter and more intense regimen, such as the one used in VanderVeen et al., i.e., 35 mg/kg once daily for 5 days (175 mg/kg cumulative), body mass was reduced, but there was no impact on skeletal muscle mass [13]. Further, Chen et al. utilised a similar regimen, i.e., 40 mg/kg once daily for 4 days (160 mg/kg cumulative), where there was evidence of a latent loss of body and skeletal muscle mass when harvested 4 days post final 5FU injection [14]. This highlights that 5FU-induced changes to muscle mass are dependent on the intensity of regimen and require a certain temporal component before they are physiologically exhibited. This concept is mirrored by functional studies, in which metronomic delivery of 5FU did not affect whole body grip strength but did when delivered in a short and intense regimen [50]. Given these data, we believe that metronomic 5FU may prime skeletal muscle with the molecular signature for myopathy without exerting a great enough challenge to result in physiological consequences.

There were some limitations to the study presented. Here, we utilised 6-week-old mice and treated them over 2 weeks, up to 8 weeks of age. Mice are generally considered to approach a plateau of their growth phase at 8 weeks of age, thus we treated them during this development period before measuring the impact of our interventions at sexual maturity. This might contribute to the outcome of the data, compared to conducting these interventions on sexually mature mice. However, both paediatric and adult cancer patients receive chemotherapy, and can each be impacted by cachexia. Further investigation is warranted in this model, with particular emphasis on a time-course study, involving both short-term and long-term experimental endpoints to investigate the temporal nature of the suite of proteins measured alongside the physiological parameters.

## 4. Materials and Methods

### 4.1. Animals

#### 4.1.1. Ethical Approval

All experimental procedures were approved by the Victoria University Animal Ethics Committee (AEETH15/006) and conformed to the Australian Code of Practice for the Care and Use of Animals for Scientific Purposes.

#### 4.1.2. Experimental Design and Treatments

Six week old male Balb/c mice were acquired from the Animal Resource Centre (ARC, Murdoch, WA, Australia) and randomly allocated to treatment groups (*n* = 8) upon arrival. Mice were housed on a 12 h light/dark cycle with ad libitum access to food (AIN-93G, Speciality Feeds, Glen Forrest, WA, Australia) and water supply throughout the experiments. Mice were administered either vehicle (VEH; 10% dimethyl sulfoxide (DMSO) in sterile water), 5-fluorouracil (5FU; 23 mg/kg (Sigma Aldrich, North Ryde, Australia) dissolved in 10% DMSO) or BGP-15 adjuvant therapy with 5FU (5FU+BGP; 15 mg/kg (BGP-15 donated by N-gene R&D, Australia) dissolved in 10% DMSO). The 5FU dose and regimen were used by us previously [51,52] and were effective at offsetting chemotoxicity symptoms associated with irinotecan treatment in skeletal muscle [12]. Treatments were administered via intraperitoneal injection 6 times over a 15 day period (i.e., on day 1, 3 5, 8, 10 and 12), resulting in a cumulative dose of 138 mg/kg and 90 mg/kg, of 5FU and BGP-15, respectively. The dosage and frequency of 5FU administration, with and without BGP-15, was based on previous published studies by us and our collaborators [12,51]. Repetitive chemotherapy dosing at set intervals (i.e., metronomic delivery) is compatible with the clinical treatment of cancer in contrast to the administration of a single bolus maximum tolerated dose that is often investigated in basic science: we have discussed this approach extensively before [48]. The selected dose is equivalent to the standard human dose per body surface area [53], and has proven efficacy in mouse models of cancer [54] and elicits toxicity in other physiological systems [51,52]. The BGP-15 dose was shown to elicit skeletal muscle protection against chemotoxicity by us previously [12,29].Animals were weighed prior to the commencement of treatment (pre), on each day of treatment and at the experimental endpoint (post).

### 4.2. Body Composition

Echo Magnetic Resonance Imaging (echoMRI) was utilized to assess the effect of 5FU treatment and BGP-15 co-therapy on body composition indices of lean and fat mass. Live mice were placed into an echoMRI body composition analyzer (EMR-150, Echo Medical Systems, Houston, TX, USA) on day 1 (pre) and day 15 (post) of the treatment protocol, as previously described [48]. Lean and fat mass was quantified via triplicate scans spaced 30 s apart and reported as the mean of these triplicate scans.

### 4.3. Surgery

At the conclusion of the treatment regimen and following the final echoMRI scan, mice were deeply anaesthetised via isoflurane inhalation, before non-recovery surgery commenced. Muscles of interest were surgically excised for ex vivo analysis in the following order: (1) right *extensor digitorum longus* (EDL) and *soleus* (SOL) muscles for the assessment of contractile properties; (2) right *tibialis anterior* (TA) muscles were harvested and immediately snap-frozen for Western blotting experiments; (3) left TA muscles were weighed and prepared for histological assessment prior to snap-freezing. The remaining tissues were then harvested (i.e., the left EDL and SOL), weighed and snap-frozen.

### 4.4. Ex Vivo Skeletal Muscle Contractile Function

Ex vivo evaluation of skeletal muscle contractile properties were performed as previously described by us [12,55], using the predominantly fast-twitch muscle, EDL and the predominantly slow-twitch muscle, SOL. Briefly, muscles were tied with 4.0 surgical silk thread, dissected from the hindlimb and attached to a transducer in individual organ baths of a Myodynamics Muscle Strip Myograph System (DMT, Aarhus, Denmark). Each organ bath was filled with Krebs solution (118 mM NaCl, 1 mM MgSO_4_.7H_2_O, 4.75 mM KCl, 1 mM Na_2_HPO, 2.5 mM CaCl_2_, 24 mM NaHCO_3_ and 11 mM glucose; pH 7.4) bubbled with carbogen (5% CO_2_ in O_2_) and maintained at a temperature of 30 ${}^{\circ }$C. Data were collected and analyzed using LabChart Pro version 8.0 software (ADInstruments, Dunedin, New Zealand). Supramaximal stimulations were delivered by flanking electrodes. Optimal length (L_o_) was established through sequential twitch contractions with incremental stretch, with peak twitch force (P_t_) derived at L_o_. Peak tetanic force (P_o_) was obtained by delivering pulse trains at 350msec and 500msec for the EDL and SOL, respectively at increasing frequencies. Twitch to tetanus ratio (P_t_/P_o_) was assessed as an indicator of elasticity/stiffness. Specific force (sP_o_) was obtained by normalising force to the physiological cross-sectional area (pCSA) as previously described [56].

### 4.5. Skeletal Muscle Histology

All histological experiments were completed as previously described [12,29]. To determine whether 5FU had atrophic effects on skeletal muscle and, subsequently, whether BGP-15 co-therapy could rescue any atrophy, we histologically assessed TA muscles which were cryopreserved in optimal cutting temperature compound (Sakura Finetek, Torrance, CA, USA). TA’s were sectioned (10 m, −20 ${}^{\circ }$C, Leica CM1950) and mounted onto glass slides, then stained with haematoxylin and eosin (H&E), for muscle fibre size analysis, and succinate dehydrogenase (SDH), as an indicator of oxidative capacity. H&E-stained slides were processed on a Zeiss Axio Imager Z2 microscope (Carl Zeiss MicroImaging GmbH, Göttingen, Germany), and imaged at 50x magnification, with analysis conducted as described previously [57]. SDH slides were processed the same way and imaged at 20x magnification. SDH images were analyzed in two ways: first, the whole cross-section of the TA was circled and the intensity density of the SDH stain was assessed using ImageJ; and second, the staining intensity of 600 individual fibres was measured using ImageJ with the maximum intensity density identified and used to determine a bottom, middle and top third. The percentage of fibres that fell into each third was determined and the data displayed as less oxidative (bottom third), more oxidative (middle third) and highly oxidative (top third).

### 4.6. Western Blotting Analyses

Western blotting was utilised to explore the effect of 5FU treatment and BGP-15 co-therapy on molecular signalling pathways surrounding cell stress, mitochondrial biogenesis and cytoskeletal structural proteins. All Western blotting protocols were completed as previously described [12]. Frozen TA muscles were homogenized using an Omni Tissue Homogenizer (TH220, Omni International, Kennesaw, GA, USA) for 20 s in ice-cold Western Immunoprecipitation Kinase (WIK) buffer (40 mM Tris, pH 7.5; 1 mM EDTA; 5 mM EGTA; 0.5% TritonX-100; 25 mM -glycerophosphate; 25 mM NaF; 1 mM Na3VO4; 10 g/ml leupeptin; and 1 mM PMSF). Muscle homogenate was centrifuged at 3500 rpm for 5 minutes at 4 ${}^{\circ }$C, before the pellet was resuspended and the muscle homogenate was frozen for future analysis. Protein concentrations were determined using a DC assay kit (Bio-Rad Laboratories, Hercules, CA, USA), to ensure equal loading on the gels. Samples were prepared with equivalent amounts of protein in either 2X SDS sample buffer (20% (*v*/*v*) glycerol; 100 mM Tris-Base, pH 6.8; 4% (*w*/*v*) SDS; 0.017% (*w*/*v*) bromophenol blue; 0.25 M dithiothreitol (DTT)), heated for 5 minutes at 95 ${}^{\circ }$C, and subjected to electrophoretic separation on 7.5–12% SDS-acrylamide gels. Following electrophoretic separation, proteins were transferred to PVDF membrane, blocked with 5% not-fat milk powder in Tris-buffered saline containing 0.1% Tween 20 (TBST) for 1 hour followed by an overnight incubation at 4 ${}^{\circ }$C with primary antibody dissolved in TBST containing either 1% BSA or 3% non-fat milk powder. The following primary antibodies were used: anti-phospho Akt^Ser473^ (1:3000; #4060; Cell Signalling Technology (CST)), anti-Akt (1:2000; #4691; CST), anti-Ankrd2 (1:1000; #11821-1-AP; Proteintech), anti--Dystroglycan (1:1000; MANDAG2 (7 D11); DSHB), anti--Sarcoglycan (1:1000; ab137101; Abcam), anti-Desmin (1:1000; #5332; CST), anti-Dystrobrevin (1:500; #610766; BD Biosciences), anti-Dystrophin (1:500; ab15277; Abcam), anti-phospho ERK1/2 (1:750; #9101; CST), anti-ERK1/2 (1:1000; #9102; CST), anti-DRP1 (1:1000; #8570; CST), anti-HSP-70 (1:1000; ADI-SPA-812; Enzo Life Sciences), anti-phospho JNK (1:750; #4668; CST), anti-JNK (1:1000; #9252; CST), anti-Laminin (1:2000; L9393; Sigma-Aldrich), anti-OPA1 (1:1000; #80471; CST), anti-phospho p38 (1:750; #4511; CST), anti-p38 (1:1000; #9212; CST), anti-phospho p65 (1:750; #3033; CST), anti-p65 (1:1000; #8242; CST), anti-PARP-1 (1:1000; #9542, CST), anti-PGC-1 (1:1000; AB3242; Sigma-Aldrich), anti-PGC-1 (1:1000; ab176328; Abcam), anti-syntrophin (1:750; MA1-745; Invitrogen), anti-talin (1:200; T3287; Merck-Millipore) and anti-TFAM (1:3000; ab131607; Abcam). After overnight incubation, membranes were washed 3 separate times for 10 minutes each in TBST and then probed with a horseradish peroxidase-conjugated secondary antibody (1:5000; anti-rabbit IgG or 1:20,000; anti-mouse IgG, Vector Laboratories) in 5% not-fat milk powder in TBST for 1 hour at room temperature. Following another set of 3 separate 10-minute washes in TBST, the blots were developed with a DARQ CCD camera mounted to a Fusion FX imaging system (Vilber Lourmat, Eberhardzell, Germany) using ECL Clarity reagent (Biorad, Hercules, CA, USA). Once images were captured, the membranes were stained with Coomassie Blue and then normalised to total protein. Densitometric measurements were carried out using FusionCAPTAdvance software (Vilber Lourmat, Eberhardzell, Germany).

### 4.7. Citrate Synthase Activity

Citrate Synthase (CS) activity was measured as a marker of mitochondrial density [58]. Homogenized TA muscles in WIK buffer (as described above) were added to the reagent cocktail (100 mM Tris Buffer, 1 mM DTNB, 3 mM Acetyl CoA) and to initiate the reaction, oxaloacetate (10 mM) was added just prior to measuring CS activity spectrophotometrically (412 nm, 25 ${}^{\circ }$C, 5 min). CS activity was calculated using the extinction coefficient of 13.6 [59] and expressed relative to muscle wet weight.

### 4.8. Cell Culture Experiments

#### 4.8.1. C2C12 Cell Culture

C2C12 myoblasts (ATCC) were cultured and maintained in Dulbecco’s modified Eagle’s medium (DMEM) supplemented with 10% foetal bovine serum (FBS), 1% antibiotic/antimycotic, 1% Glutamax, and 1 mM sodium pyruvate (all cell culture reagents were purchased from Gibco Invitrogen, Carlsbad, CA, USA). Cells were incubated at 37 ${}^{\circ }$C with 5% CO_2_. To induce differentiation, cells were plated at high confluence (~90%) and changed to differentiation medium (supplemented DMEM containing 2% horse serum). Cells were maintained in differentiation medium for 4–5 days to form myotubes before commencing drug treatments. Differentiated myotubes were treated with vehicle control (VEH; DMSO), BGP-15 control (BGP; 100 mM), 5FU (1 mM) and the 5FU+BGP combination for 24 h prior to assays and protein collection.

#### 4.8.2. Protein Collection

Cells were lysed and collected for Western blotting in radio immunoprecipitation buffer (RIPA; 1 mM EDTA, 0.5 mM EGTA, 10 mM Tris-HCl, 140 mM sodium chloride, 10% sodium deoxycholate, and 1% triton-X 100) containing protease and phosphatase inhibitors (Sigma Aldrich, St. Louis, MO, USA). The lysates were centrifuged at 13,000 rpm at 4 ${}^{\circ }$C for 30 minutes. The supernatants were collected and stored at −80 ${}^{\circ }$C and the pellets discarded. Western blotting was performed as described above.

#### 4.8.3. Resazurin Cell Viability Assay

Confluent myotubes were treated with drugs of interest for up to 24 h in differentiation medium prior to resazurin viability assay (Sigma Aldrich, St. Louis, MO, USA) measurements. The resazurin assay solution was prepared at a 1:10 dilution of resazurin in DMEM differentiation medium. Once the dye was added the plates were shielded from light and stored at 37 ${}^{\circ }$C with 5% CO_2_ for two hours. Following the incubation period, the supernatant was transferred to an opaque 96 well plate for fluorometric reading (at 560 nm; Varioskan Flash plate reader) using SkanIt RE software (Thermo Fisher, Waltham, MA, USA). Plates containing myotubes were rinsed with ice cold PBS and fixed with 100% methanol, followed by Diff-Quick staining (Histolabs, Kew East, Australia). Plates were left to dry overnight prior to imaging with an Olympus IX81 microscope (Olympus, Tokyo, Japan) to observe morphological changes.

### 4.9. Statistics

Data are presented as the mean ± standard error of the mean (SEM). Data were analyzed using Graphpad prism v9 (GraphPad Software, San Diego, CA, USA). A one-way ANOVA was utilised to detect treatment differences for parametric data, while a two-way repeated measures ANOVA was used to detect differences between treatment and stimulation frequency/time for force–frequency relationships. Tukey’s post hoc test was utilised for multiple comparisons testing, with an -value of 0.05 considered significant.

## 5. Conclusions

This is the first study to demonstrate that metronomic delivery of 5FU reduces the expression of desmin and dystrophin, which is not affected by BGP-15. However, BGP-15 mitigated the 5FU-induced increase in p38 and NF-B phosphorylation, which was associated with BGP-15 increasing mitochondrial density and fusion dynamics. Paradoxically, metronomic delivery of 5FU did not impact body composition indices, nor skeletal muscle mass and function. These findings suggest that the metronomic delivery of 5FU may prime skeletal muscle with a molecular signature for myopathy but does not exert a great enough challenge to result in physiological consequences within the treatment duration employed in this study. Overall, these data highlight novel mechanisms surrounding the impact of 5FU treatment and BGP-15 adjuvant therapy on skeletal muscle, supporting the need for further investigation, particularly in cancer-burdened mice to enhance the translational potential of the dataset.

## Data Availability

There are no archived digitally archived data sets associated with this manuscript.

## Supplementary Materials

The following are available online at https://www.mdpi.com/article/10.3390/ph14050478/s1, Table S1: Organ to body mass ratios, Figure S1: Full-length Western blot images relating to the skeletal muscle homogenate data presented in Figure 3, Figure S2: Densitometry summary data from representative Western blot images, Table S2 Densitometry summary data from representative Western blot images, Figure S3: Full-length Western blot images relating to the C2C12 myotube cell lysate data presented in Figure 6.
