# Supplementary material for: Metronomic 5-Fluorouracil Delivery Primes Skeletal Muscle for Myopathy but Does Not Cause Cachexia
Source: Pharmaceuticals (Basel). 2021 May 17;14(5):478. doi: 10.3390/ph14050478 (PMC8156038; doi:10.3390/ph14050478)
Supplement: Supplementary file 1 [file pharmaceuticals-14-00478-s001.zip › pharmaceuticals-1185223-supplementary.pdf]

Article- Supplementary Information

Metronomic 5-fluorouracil delivery primes skeletal muscle for myopathy but does not cause cachexia

Dean G. Campelj [1,2], Cara A. Timpani[1,2,3], Tabitha Cree[1,2], Aaron C. Petersen[1,2], Alan Hayes[1,2,3], Craig A. Goodman[1,2]* and Emma Rybalka[1,2,3]*

[1] Institute for Health and Sport, Victoria University, Melbourne, Victoria, Australia; dean.campelj@live.vu.edu.au; cara.timpani@vu.edu.au; tabitha.cree@live.vu.edu.au; aaron.petersen@vu.edu.au

[2] Australian Institute for Musculoskeletal Science (AIMSS), Inherited and Acquired Myopathy Program, Victoria University, St Albans, Victoria, Australia; emma.rybalka@vu.edu.au

[3] Department of Medicine - Western Health, Melbourne Medical School, The University of Melbourne, Melbourne, Victoria, Australia; alan.hayes@vu.edu.au

[4] Centre for Muscle Research (CMR), Department of Physiology, The University of Melbourne, Parkville, Victoria, Australia; craig.goodman@unimelb.edu.au

* Correspondence: emma.rybalka@vu.edu.au, craig.goodman@unimelb.edu.au

Table. S1 Organ to body mass ratios. Various organs from all treatment groups were surgical excised and weighed at the end of the treatment period. *n*= 7-8.

	Organ/Body Mass Ratio (mg/g)		
	**VEH**	**5FU**	**5FU+BGP**
**Kidney**	8.70 ± 0.26	8.96 ± 0.26	8.40 ± 0.21
**Spleen**	3.82 ± 0.07	4.14 ± 0.18	3.87 ± 0.11
**Liver**	54.18 ± 0.83	54.5 ± 2.00	49.61 ± 1.68
**Lungs**	7.05 ± 0.07	7.09 ± 0.16	7.17 ± 0.22
**Heart**	5.21 ± 0.10	5.46 ± 0.45	5.33 ± 0.22

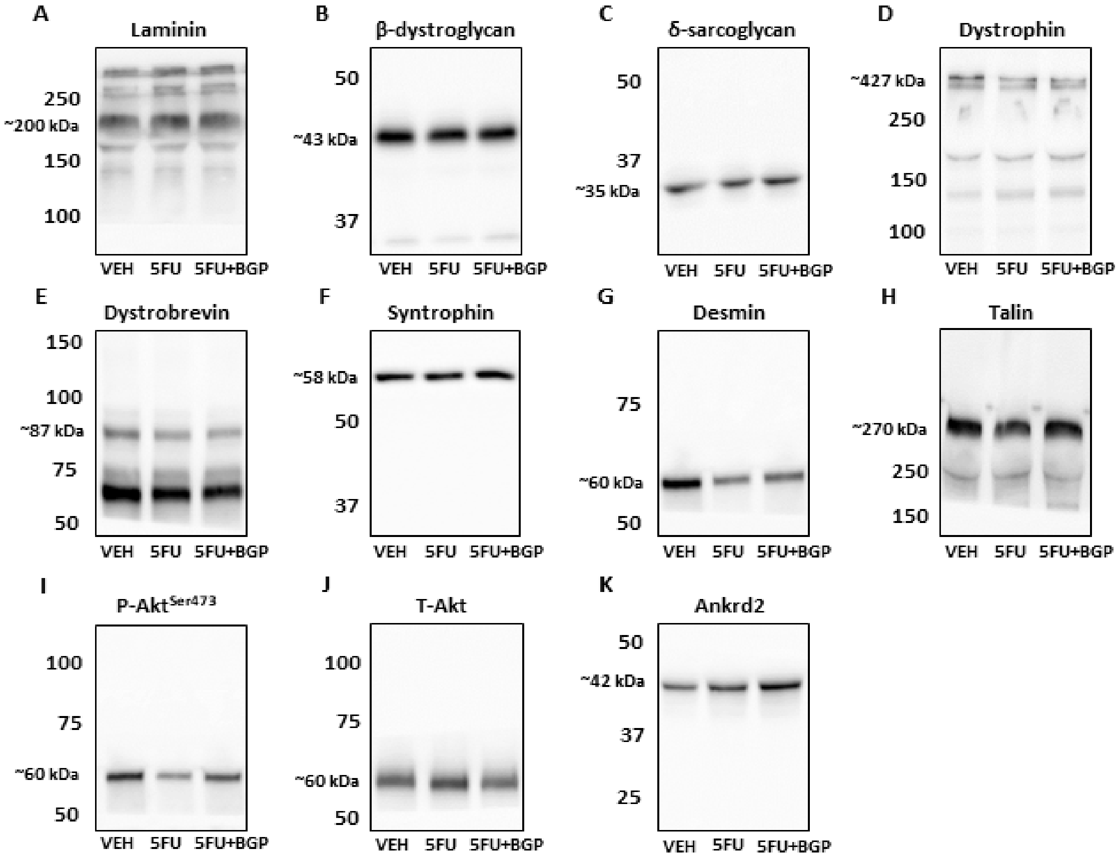

Fig. S1 Full-length Western blot images relating to the skeletal muscle homogenate data presented in Fig. 3. After transferring the protein from the gel to the PVDF membrane, membranes were cut horizontally to allow for probing with multiple primary antibodies on a single membrane/gel. Subsequently, the above images display the largest available vertical membrane area probed with each respective primary antibody.

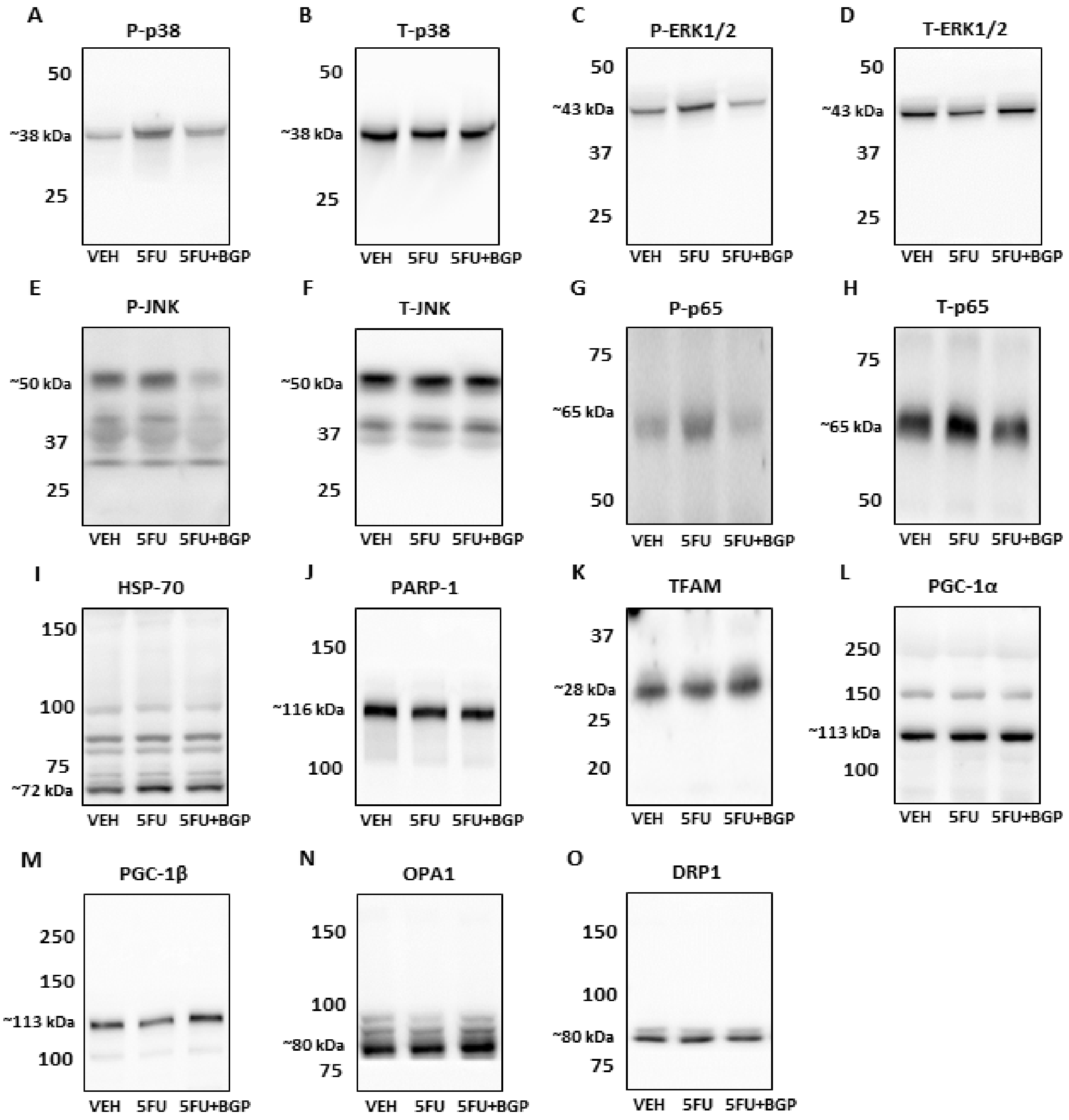

Fig. S2 Full-length Western blot images relating to the skeletal muscle homogenate data presented in Fig. 4 and 5. After transferring the protein from the gel to the PVDF membrane, membranes were cut horizontally to allow for probing with multiple primary antibodies on a single membrane/gel. Subsequently, the above images display the largest available vertical membrane area probed with each respective primary antibody.

Table. S2 Densitometry summary data from representative Western blot images. Data is presented as integrated density relative to total protein (in arbitrary units, i.e. (a.u.)) for all antibodies analysed.

	Protein/Total Protein (a.u.)				Protein/Total Protein (a.u.)		
**Target Protein**	**VEH**	**5FU**	**5FU+BGP**	**Target Protein**	**VEH**	**5FU**	**5FU+BGP**
P-Akt^{Ser473}	36.14	16.18	27.75	Laminin	43.59	45.55	42.90
T-Akt	103.68	123.27	131.06	OPA1	22.14	23.63	27.59
Ankrd2	16.21	17.65	27.12	P-p38	20.31	53.45	31.70
Desmin	7.26	5.08	4.31	T-p38	94.01	96.11	105.17
DRP1	15.35	16.42	15.35	P-p65	4.96	6.27	4.67
Dystrobrevin	3.74	3.90	4.73	T-p65	17.43	14.23	16.02
β-Dystroglycan	16.22	14.70	14.55	PARP-1	45.07	38.80	36.58
Dystrophin	9.55	6.56	5.95	PGC-1α	14.92	17.37	16.15
P-ERK1/2	28.08	45.25	17.20	PGC-1β	26.16	30.40	32.06
T-ERK1/2	81.14	65.68	89.88	δ-Sarcoglycan	8.74	7.18	9.07
HSP-70	6.10	7.25	6.28	Syntrophin	10.81	10.47	11.86
P-JNK	18.04	18.54	9.78	Talin	36.50	33.60	31.84
T-JNK	40.68	38.06	41.52	TFAM	12.92	12.77	15.30

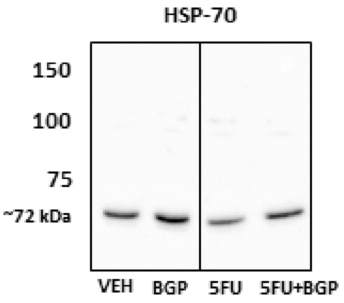

	Protein/Total Protein (a.u.)			
Target Protein	VEH	BGP	5FU	5FU+BGP
HSP-70	3.67	5.52	2.87	4.24

Fig. S3 Full-length Western blot images relating to the C2C12 myotube cell lysate data presented in Fig. 6. After transferring the protein from the gel to the PVDF membrane, membranes were cut horizontally to allow for probing with multiple primary antibodies on a single membrane/gel. Subsequently, the above images display the largest available vertical membrane area probed with each respective primary antibody.
